# Cooling of male rat skeletal muscle during endurance‐like contraction attenuates contraction‐induced PGC‐1α mRNA expression

**DOI:** 10.14814/phy2.15867

**Published:** 2023-11-14

**Authors:** Daisuke Hoshino, Ryota Wada, Yutaro Mori, Reo Takeda, Yudai Nonaka, Ryotaro Kano, Ryo Takagi, Yutaka Kano

**Affiliations:** ^1^ Bioscience and Technology Program, Department of Engineering Science The University of Electro‐Communications Chofu, Tokyo Japan; ^2^ Institute of Liberal Arts and Science, Kanazawa University Kanazawa Japan; ^3^ Ritsumeikan Global Innovation Research Organization Ritsumeikan University Kusatsu, Shiga Japan

**Keywords:** glycogen, lactate, signal molecules, temperature

## Abstract

This study aimed to determine effects of cooling on contraction‐induced peroxisome proliferator‐activated receptor γ coactivator‐1α (PGC‐1α) and vascular endothelial growth factor (VEGF) gene expression, phosphorylations of its related protein kinases, and metabolic responses. Male rats were separated into two groups; room temperature (RT) or ice‐treated (COLD) on the right tibialis anterior (TA). The TA was contracted isometrically using nerve electrical stimulation (1‐s stimulation × 30 contractions, with 1‐s intervals, for 10 sets with 1‐min intervals). The TA was treated before the contraction and during 1‐min intervals with an ice pack for the COLD group and a water pack at RT for the RT group. The muscle temperature of the COLD group decreased to 19.42 ± 0.44°C (*p* < 0.0001, −36.4%) compared with the RT group after the experimental protocol. An increase in mRNA expression level of *PGC‐1α*, not *VEGF*, after muscle contractions was significantly lower in the COLD group than in the RT group (*p* < 0.0001, −63.0%). An increase in phosphorylated AMP‐activated kinase (AMPK) (*p* = 0.0037, −28.8%) and a decrease in glycogen concentration (*p* = 0.0231, +106.3%) after muscle contraction were also significantly inhibited by cooling. Collectively, muscle cooling attenuated the post‐contraction increases in *PGC‐1α* mRNA expression coinciding with decreases in AMPK phosphorylation and glycogen degradation.

## 1 INTRODUCTION

Prolonged exercise training enhances oxidative metabolic capacity by inducing mitochondrial biogenesis and angiogenesis in skeletal muscles. Such chronic adaptations are based on molecular responses induced by acute exercises, such as activation of signal transduction by metabolic, ionic, and mechanical stimuli, and transient changes in gene expression (Egan & Sharples, 2022). For instance, acute exercise transiently increases the activity of signaling molecules such as AMP‐activated kinase (AMPK), which is activated by increased metabolic demands, calcium/calmodulin‐dependent protein kinase II (CaMKII), which is activated by intracellular calcium, and p38 mitogen‐activated protein kinase (p38), which is activated by mechanical stimuli and/or reactive oxygen species (Egan et al., 2010; Gibala et al., 2009; Perry & Hawley, 2018). Activation of these kinases increases the level of peroxisome proliferator‐activated receptor gamma coactivator 1‐alpha (PGC‐1α), which is required for mitochondrial biogenesis (Egan et al., 2010; Perry & Hawley, 2018; Zhang et al., 2014). Repeated transient elevations in PGC‐1α are thought to increase proteins related to mitochondrial, glucose, and fat metabolism, hence remodeling skeletal muscle oxidative metabolism (Perry et al., 2010). PGC‐1α also modulates exercise‐induced angiogenesis by its effects on vascular endothelial growth factor (VEGF) (Chinsomboon et al., 2009). It is important to explore effective physical intervention techniques that result in greater activation of its upstream metabolic signaling molecules, as well as an increase in *PGC‐1α* mRNA expression after exercise for more effective exercise prescription to improve muscle oxidative capacity.

One intervention that may influence the increase in *PGC‐1α* mRNA expression after exercise is a cooling stimulus. For instance, Wakabayashi et al. stated that a 30‐min cycling exercise with human thighs lowered to 22°C using a cool water‐circulating pad enhanced the glycolytic metabolism in skeletal muscle and increased blood lactate concentration more than the exercise at normal temperature (Wakabayashi et al., 2018). Local thigh cooling to roughly 20°C has also been shown to increase blood noradrenaline levels after exercise in humans (Joo et al., 2016). Since these metabolic stimuli such as increased glycolytic demands and beta‐2 adrenergic stimulations have been shown to increase *PGC‐1α* mRNA expression (Egan et al., 2010; Miura et al., 2007; Tadaishi et al., 2011), exercise with local cooling may enhance subsequent increases in *PGC‐1α* mRNA expression. On the other hand, low temperature reduced the maximal tension of muscular contraction. For example, experiments with rodent muscles have revealed that the maximum tension of isometric contraction is lowered by 10%–20% at a muscle temperature of 20–22°C compared to 35°C (Stephenson & Williams, 1981; Truong et al., 1964; Westerblad et al., 1997). As postexercise *PGC‐1α* mRNA expression increases in an exercise intensity‐dependent manner (Egan et al., 2010; Tobina et al., 2011), muscular contraction with cooling may decrease the subsequent increase in *PGC‐1α* mRNA expression by lowering the muscle tension.

Recently, it was found that the response of *PGC‐1α* mRNA expression to endurance exercise in humans was reduced when the muscle temperature was reduced to 29°C compared to exercise at room temperature (RT) (Meister et al., 2021). However, the muscle temperature (29°C) in their study was approximately 10°C higher than that in the prior study (20°C), in which lactate production was enhanced during exercise (Wakabayashi et al., 2018). Furthermore, they examined only mRNA expression, and did not investigate metabolic parameters such as muscle glycogen and lactate concentrations or signaling molecule phosphorylations that might be important in *PGC‐1α* mRNA expression after exercise (Meister et al., 2021). Therefore, the purpose of this study was to determine the effects of local cooling to a muscle temperature of approximately 20°C, on *PGC‐1α* and *VEGF* mRNA expressions and their upstream metabolic responses and signaling molecules after muscle contraction. We used low‐frequency (20 Hz) endurance‐like muscle contraction (Hamada et al., 2004; Yamada et al., 2021). Specifically, we evaluated *PGC‐1α* and *VEGF* mRNA expressions, muscle glycogen and lactate concentrations, and phosphorylations of signaling molecules immediately or 3 h after a muscular contraction in rats utilizing 20 Hz electrical stimulation on skeletal muscle at approximately 20°C and RT.

## 2 MATERIALS AND METHODS

### 2.1 Animals

Thirty‐one 11‐week‐old male Wistar rats (Japan SLC Inc., Shizuoka, Japan) were used. The rats were divided into two groups; control RT and ice‐treated (COLD). All animals were maintained at 22–24°C with a 12:12‐h light–dark cycle and were allowed food and water ad libitum. All experiments were conducted under the guidelines established by the Physiological Society of Japan and were approved by the University of Electro‐Communications Institutional Animal Care and Use Committee (Tokyo, Japan; Approval No. A39).

### 2.2 Preparation of muscle contraction and temperature treatment

Under 2% isoflurane (Pfizer Japan, Tokyo, Japan) anesthesia, the animals were placed supine on a heated plate set at 37°C. A razor was used to shave the area around the tibialis anterior muscle (TA). A small incision was made around the peroneal head of the right leg, and a stainless electrode was attached to the deep peroneal nerve for electrical stimulation. In the ankle, a small hole was made between the TA and extensor digitorum longus tendons. A temperature sensor (BAT‐10: Physitemp Instruments, Clifton, NJ) was placed through the opening to the middle of the TA surface under the fascia. Rats were positioned in a supine position with their right foot on a footplate (the ankle joint angle was set at 90°). In addition, a temperature sensor was also placed in the rectum. Muscle and rectal temperatures were monitored every minute.

### 2.3 Muscle contraction

The right TA was electrically contracted under isoflurane anesthesia by stimulation to the peroneal nerve using the stainless electrode (Unique Medical, Tokyo, Japan), and an electric stimulator (Model NS‐101, Unique Medical, Tokyo, Japan). The right leg was fixed to an exercise load device (RU‐72 model: Motomura Systems, Tokyo, Japan). The TA was isometrically contracted (1‐s stimulation × 30 contractions, with 1‐s gaps between contractions, for 10 sets with 1‐min rest intervals) with the voltage (10–14 V) and stimulation frequency (20 Hz, stimulus duration 700 ms). The right ankle joint was set at 90° and the muscle tension generated during isometric contraction was monitored using a strain gauge that was incorporated into the plate fixing the foot. A muscle tension of 100 Hz was evaluated as an indicator of maximum isometric tension prior to the first muscular contraction. The right leg was the stimulated leg (STM) and the left leg was the control leg (CON). All data on muscle tension are normalized by peak torque of 100 Hz in each animal at RT. The total integral was an area under the curve calculated by % of 100 Hz × s. The integral of >50% of 100 Hz was an area under the curve more than 50% of 100 Hz. Half‐rise time was the duration from the onset of electrical stimulation until 50% of the maximum tension was reached in the first muscle contraction. Half‐fall time was the duration from the end of electrical stimulation until 50% of the maximum tension was reached in the first muscle contraction.

### 2.4 Temperature treatment

For 3 min before the muscular contraction, the right TA was treated with an ice pack (crushed ice) for the COLD group and a room‐temperature (22–24°C) water pack for the RT group using polyethylene bags. The identical interventions were carried out during each 1‐min period between the sets. Muscle and rectal temperatures were measured during the experiment every minute.

### 2.5 Muscle collection

Both TA muscles were harvested immediately or 3 h after the muscle contraction under isoflurane anesthesia. Rats collecting muscle 3 h after the muscle contraction were maintained at rest for 3 h. All muscle samples were immediately frozen in liquid nitrogen and kept at −80°C. After the muscle collection, animals were euthanized by an overdose of isoflurane.

### 2.6 Western blot

Muscles collected immediately after the muscle contraction were used for western blot. The middle portion of TA (50 mg) was homogenized in the cold RIPA lysis buffer (0.5 M Tris–HCl, 1.5 M NaCl, 2.5% deoxycholic acid, 10% NP‐40, 10 mM EDTA, pH 7.4; 20–188, Millipore, Burlington, MA) containing protease inhibitor cocktail (11836170001, Sigma‐Aldrich, St. Louis, MO) and phosphatase inhibitors (04906837001, PhosSTOP; Roche, Basel, Switzerland). The supernatants were collected after homogenized samples were centrifuged for 1500 g for 15 min at 4°C. The total protein concentration was determined using the BCA Protein Assay Kit (23227, Pierce, Rockford, IL). An identical amount of protein (10 μg) was added to 10% SDS‐PAGE gels and separated. The wet transfer was used to transfer proteins to polyvinylidene difluoride membranes (100 V, 80 min). The membranes were blocked with 5% skim milk diluted in Tris‐buffered saline containing 0.1% Tween‐20 (TBS‐T) for 1 h at RT. The membranes were incubated overnight at 4°C with primary antibodies diluted 1:1000 in TBS‐T containing 5% skim milk or 5% BSA (phosphorylated (p)AMPKα [Thr172, #2535, Cell Signaling Technology (CST), Danvers, MA], pp38 [Thr180/Tyr182, #9211, CST], pCAMKII [Thr286, #12716, CST], AMPK [#5831, CST], p38 [#9212, CST], and CaMKII [#4436, CST]). After a 1‐h incubation with secondary antibody diluted 1:3000 in TBS‐T (anti‐rabbit IgG, #7074, CST), the bands were visualized by a chemiluminescence reagent (RPN3243, Cytiva, Tokyo, Japan), and quantified by densitometry (LAS‐4000, Fujifilm, Tokyo, Japan). Ponceau S staining was performed to confirm the equal loading of samples.

### 2.7 Real‐time PCR

Muscles collected 3 h after the muscle contraction were used for real‐time PCR. Three hours after the muscle contraction is appropriate to evaluate *PGC‐1α* and *VEGF* mRNA expression (Leick et al., 2009; Wright et al., 2007). The middle portion of TA (50 mg) was homogenized in cold TRIzol reagent (15596018, Thermo Fisher Scientific, Waltham, MA) using the beads crusher (uT‐01, Taitec, Saitama, Japan). Following homogenization, total RNA was extracted using the NucleoSpin RNA kit (740955, Takara, Shiga, Japan), and RNA concentration was determined using NanoDrop Lite (Thermo Fisher Scientific). Sufficient mRNA concentrations were obtained (350.0–856.7 ng/μL) and 500 ng of total mRNA was performed subsequent reverse transcription using high‐capacity RNA‐to‐cDNA Kit (4387406, Thermo Fisher Scientific). Real‐time polymerase chain reaction (PCR) was carried out in duplicate using the StepOne System (Thermo Fisher Scientific) and SYBR Green (A25780, Thermo Fisher Scientific). 18S ribosomal RNA was used as an internal control. The fold changes were calculated on the basis of the ΔΔCt method. The following primers were purchased from Takara and used: 18S ribosomal RNA, forward, 5′‐AAGTTTCAGCACATCCTGCGAGTA‐3′, and reverse, 5′‐TTGGTGAGGTCAATGTCTGCTTTC‐3′; *PGC‐1α*, forward, 5′‐ACCGTAAATCTGCGGGATGA‐3′, and reverse, 5′‐AGTTTCATTCGACCTGCGTAAAGTA‐3′; *VEGF*, forward, 5′‐TCCTGCAGCATAGCAGATGTGA‐3′, reverse, 5′‐CCAGGATTTAAACCGGGATTTC‐3′, beta‐2 microglobulin, forward, 5′‐CCTGGCTCACACTGAATTCACAC‐3′, and reverse, 5′‐AACCGGATCTGGAGTTAAACTGGTC‐3′.

### 2.8 Muscle glycogen and lactate concentration

Muscles collected immediately after the muscle contraction were used for muscle glycogen and lactate concentration. The middle portion of TA (50 mg) was homogenized 25 times (vol/wt) with 0.3 M perchloric acid. The sample (25 μL) was transferred to 500 μL of HCl and boiled for 2 h. After the acid hydrolysis, the samples were and neutralized by adding 500 μL of 1 M NaOH. Using this sample, glucose concentration was assayed using the enzymatic methods (716251, Roche, Basel, Switzerland). Using same neutralized samples, lactate concentration was assayed using the commercial kit (ab65331, Abcam, Cambridge, UK).

### 2.9 Statistical analysis

All data were presented as the mean ± standard deviation. Protein and mRNA values were normalized by the mean of CON legs in RT animals. A two‐way repeated measures analysis of variance (ANOVA) was performed to examine the differences (temperature treatment and time or stimulation). When the interaction was significant, post hoc comparisons were performed using the Tukey test. Two‐tailed unpaired Student's *t*‐test was used to assess the remaining data. All statistical analyses were performed using GraphPad Prism version 9 Software (GraphPad, San Diego, CA, USA). *p* < 0.05 was set as the significance threshold.

## 3 RESULTS

### 3.1 Muscle and rectal temperatures

**FIGURE 1:**
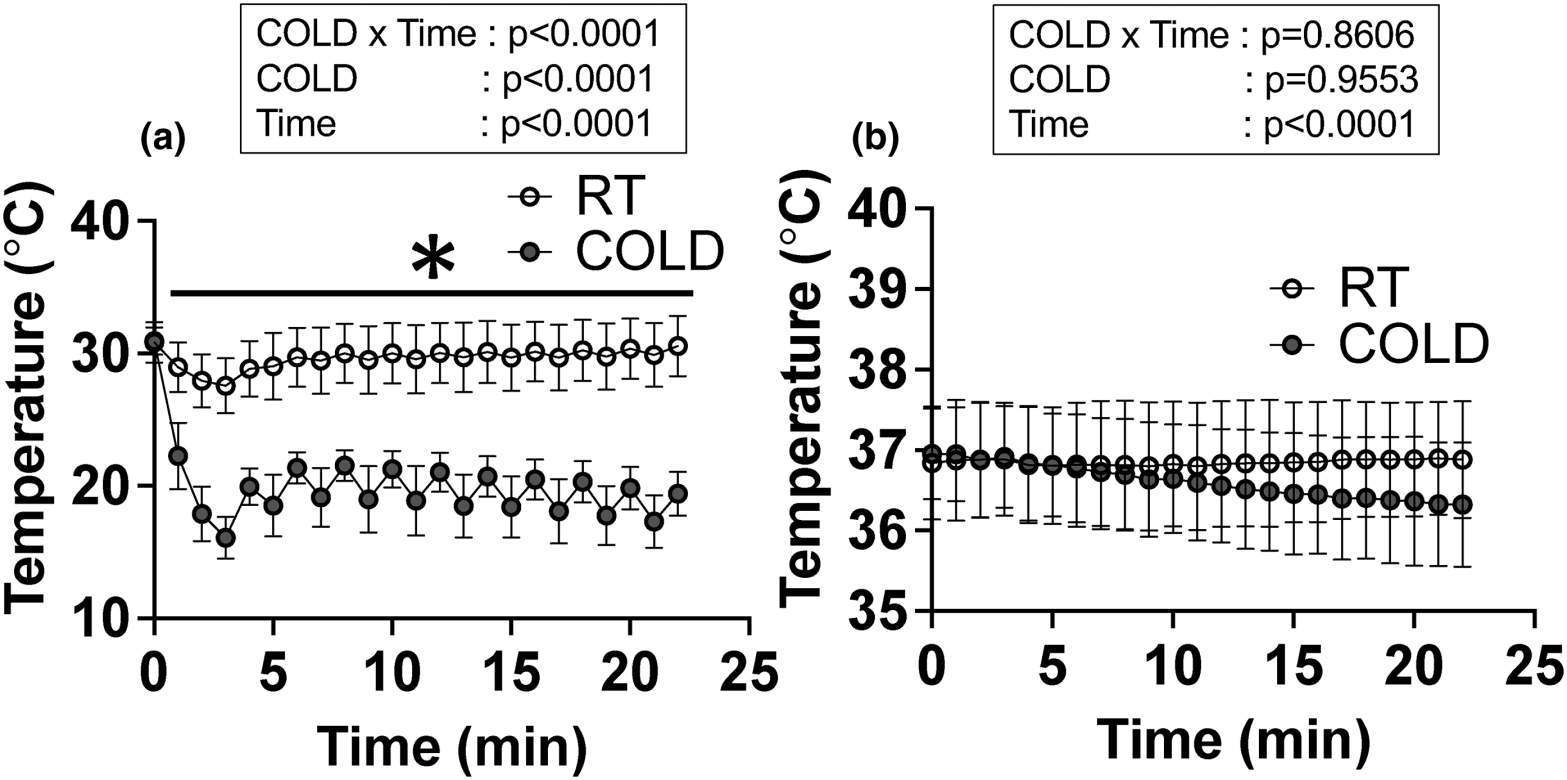
(a) Muscle and (b) rectal temperature. *N* = 14 rats, Data are mean ± SD. *p < 0.0001, RT versus COLD at 1–22 min. 0–3 min was the temperature treatment phase. After the 3 min, 1‐min muscle contraction protocol, and 1‐min temperature treatment was repeated 10 times.

Muscle and rectal temperatures are shown in Figure 1a,b, respectively. For muscle temperature, a significant main effect of COLD (*p* < 0.0001), time (*p* < 0.0001), and a significant interaction of those factors (*p* < 0.0001) were detected in the two‐way ANOVA. The muscle temperature of the COLD group was significantly lower than that of the RT group during the experimental protocol (*p* < 0.0001, RT vs. COLD at 1–22 min using a post hoc test). The muscle temperature of the RT group was 30.57 ± 0.58°C and that of the COLD group was 19.42 ± 0.44°C at the end of the experimental protocol (−36.4%). There was a significant main effect of time (*p* < 0.0001) but the rectal temperature was not significantly altered between the two groups during the experimental protocol. After the experimental protocol, the rectal temperature of the COLD group was 36.88 ± 0.18°C and that of the RT group was 36.32 ± 0.20°C.

### 3.2 Muscle tension

**FIGURE 2:**
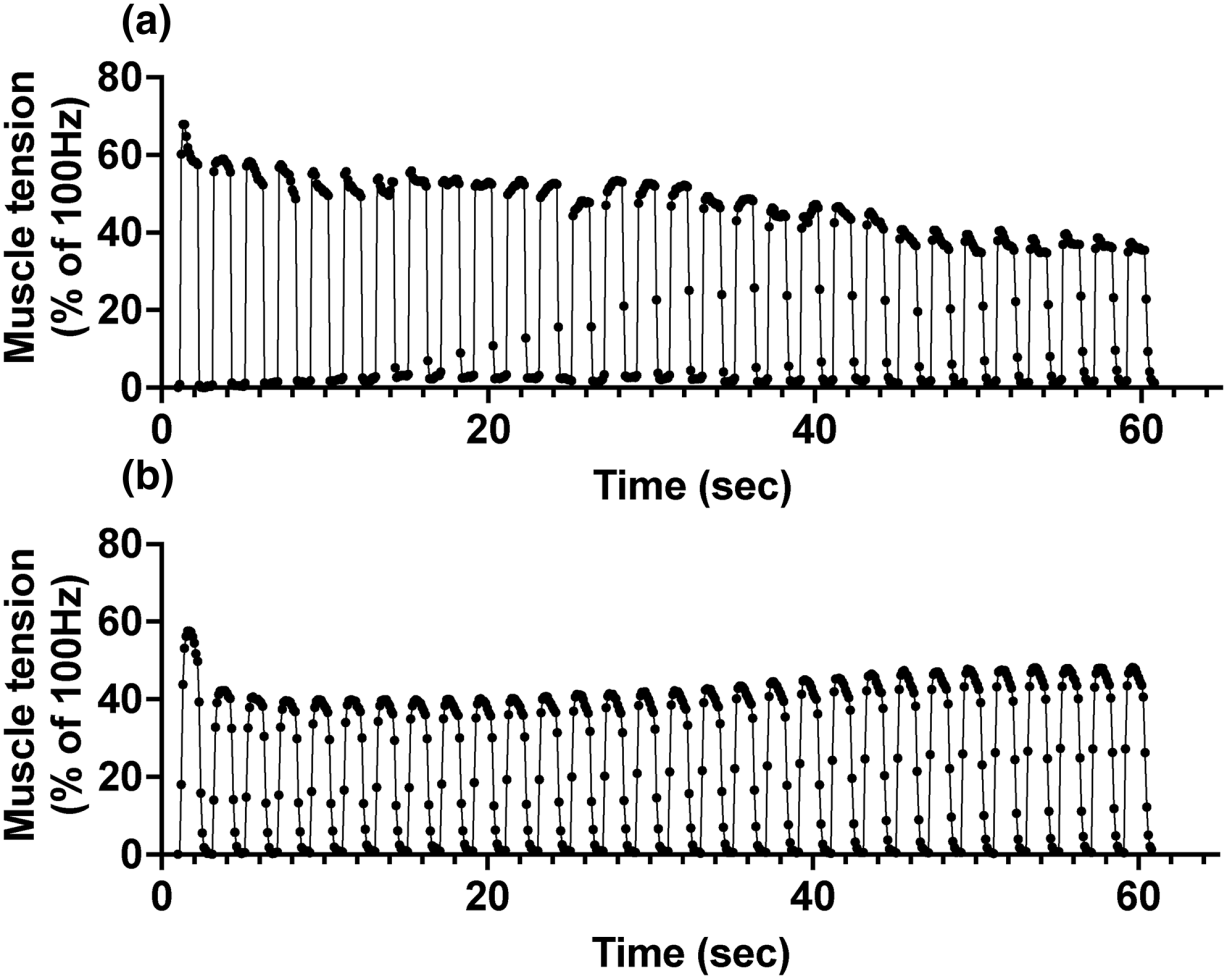
The representative data of muscle tension during the first set of muscle contractions. (a) RT and (b) COLD group.

**FIGURE 3:**
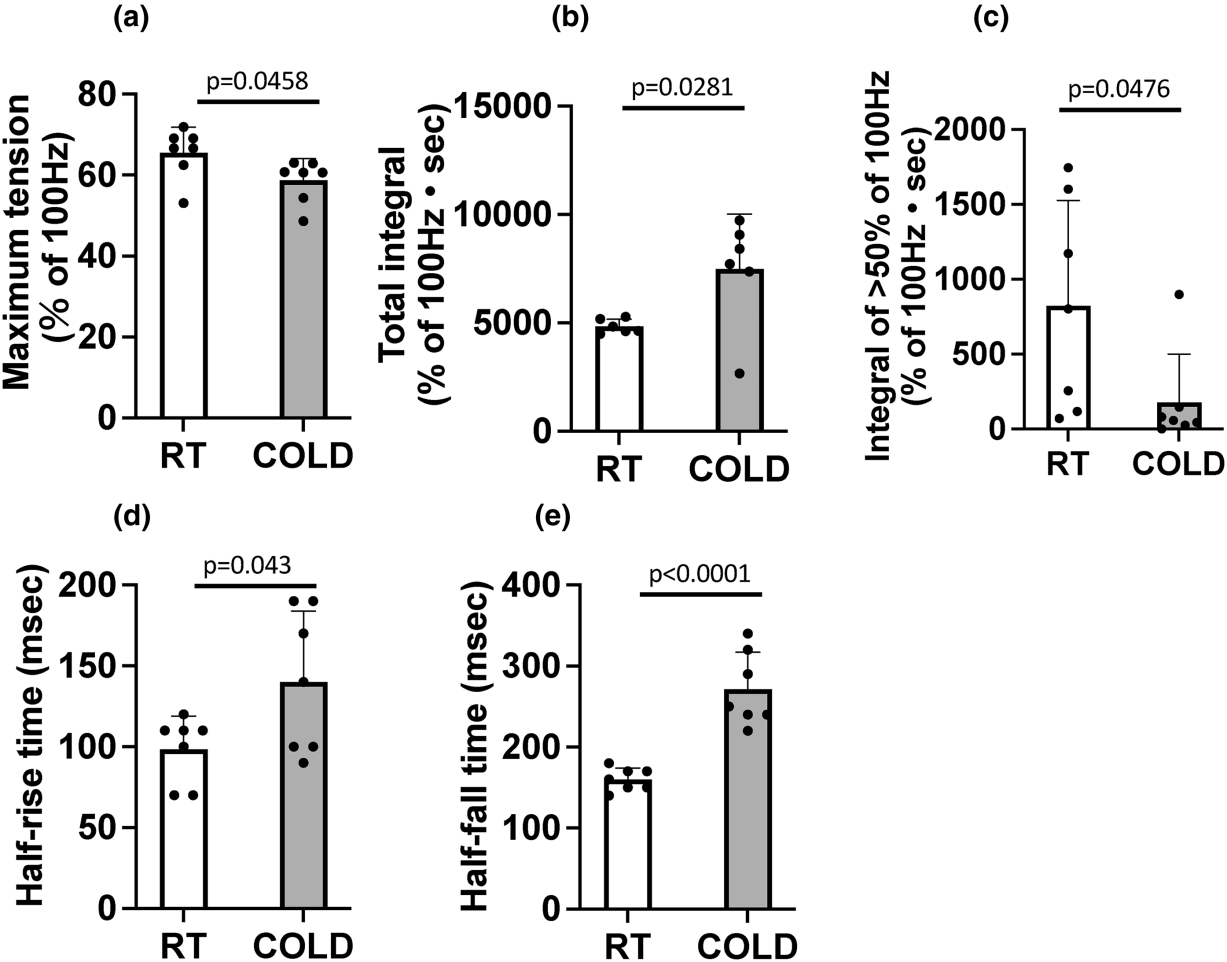
(a) Maximum tension at 20 Hz (% of 100 Hz at room temperature). (b) Total integral, and (c) Integral of >50% of 100 Hz muscle tension during first–tenth sets of muscle contractions. (d) Half‐rise time and (e) half‐fall time of the first muscle contraction of the first set. White (RT), gray (COLD) group. *N* = 6 or 7 rats, Data are mean ± SD.

Representative data of muscle tension for first set of muscle contractions is depicted in Figure 2 (a: RT, b: COLD). Peak tetanus torque tension of 100 Hz at RT prior to the temperature treatment was not significantly different between the two groups (RT: 35.31 ± 1.89 mNm, COLD: 36.03 ± 1.45 mNm). All data on muscle tension are expressed as relative values normalized by peak torque of 100 Hz in each rat. Maximum tension utilizing 20 Hz stimulation of the COLD group was significantly lower than the RT group (Figure 3a, *p* = 0.0458, −9.7%). The total integral was significantly higher in the COLD group (Figure 3b, *p* = 0.0281, +35.4%) but the integral of >50% of 100 Hz tension was lower in the COLD than in the RT group (Figure 3c, *p* = 0.0476, −78.2%). We calculated half‐rise time and half‐fall time of first muscle contraction of first set as an index of the velocity of contraction and relaxation, respectively. Half‐rise time and half‐fall time were significantly longer in the COLD group than in the RT group (Figure 3d, half‐rise time + 42.0%, *p* = 0.043 and Figure 3e, half‐fall time + 69.6%, *p* < 0.0001).

### 3.3 Muscle glycogen and lactate concentration

**FIGURE 4:**
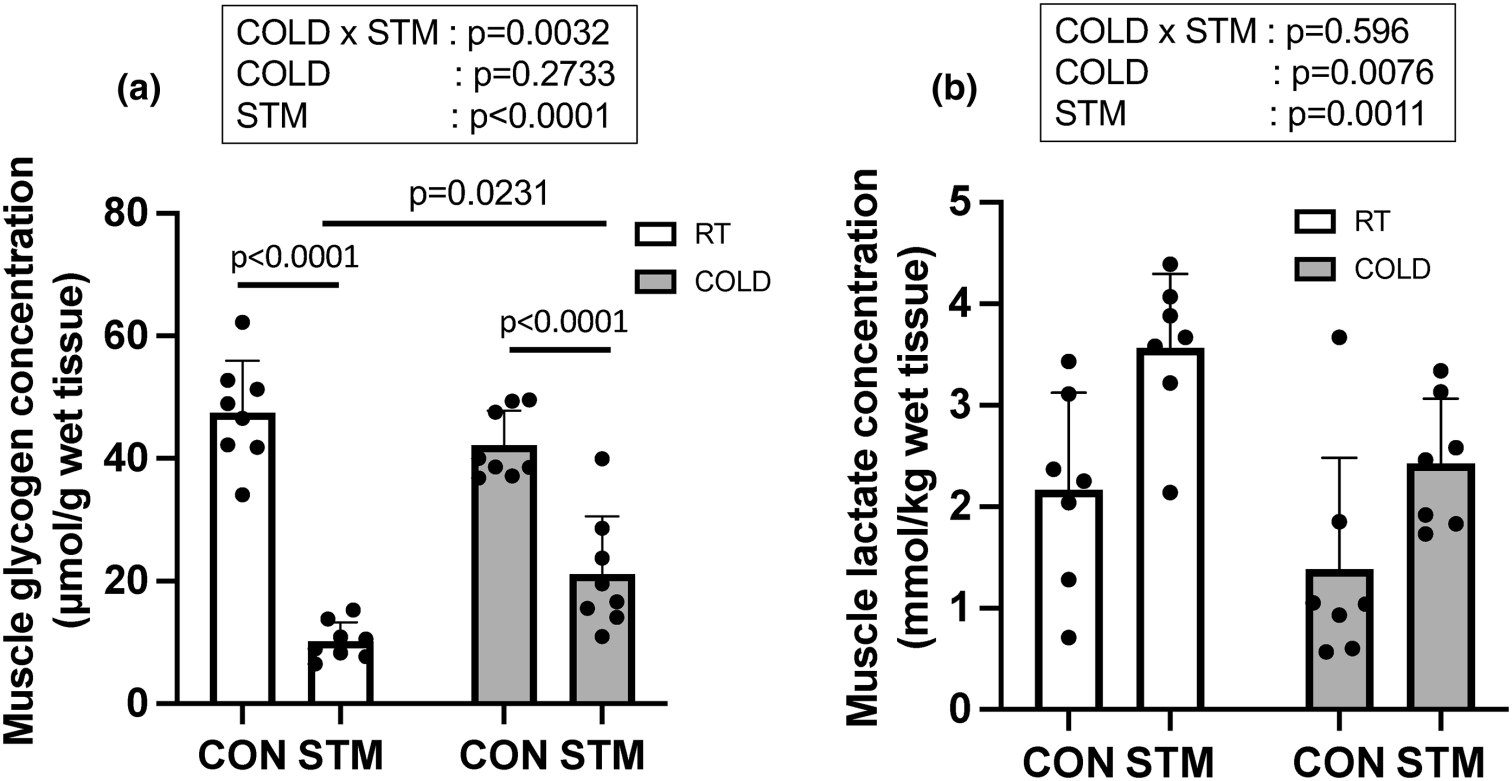
(a) Muscle glycogen and (b) lactate concentration immediately after the last muscle contraction. *n* = 7 or 8 muscles, Data are mean ± SD.

For muscle glycogen concentration, a significant main effect of STM (*p* < 0.0001), and a significant interaction of COLD versus STM (*p* = 0.0032) were detected in the two‐way ANOVA (Figure 4a). Muscle contraction significantly decreased glycogen concentration in RT (*p* < 0.0001, −78.3%) and COLD (*p* < 0.0001, −49.8%) groups. Glycogen concentration was significantly higher in the COLD group than in the RT group (*p* = 0.0231, +106.1%). Lactate concentrations were significantly increased after the muscle contraction in both groups (Figure 4b, *p* = 0.0011 main effect of STM, RT: +64.2%, COLD: 74.9%) and were significantly decreased with the cold treatment (*p* = 0.0076 main effect of COLD, CON: −36.0%, STM: −31.9%).

### 3.4 Phosphorylated AMPK, CaMKII and p38 levels

**FIGURE 5:**
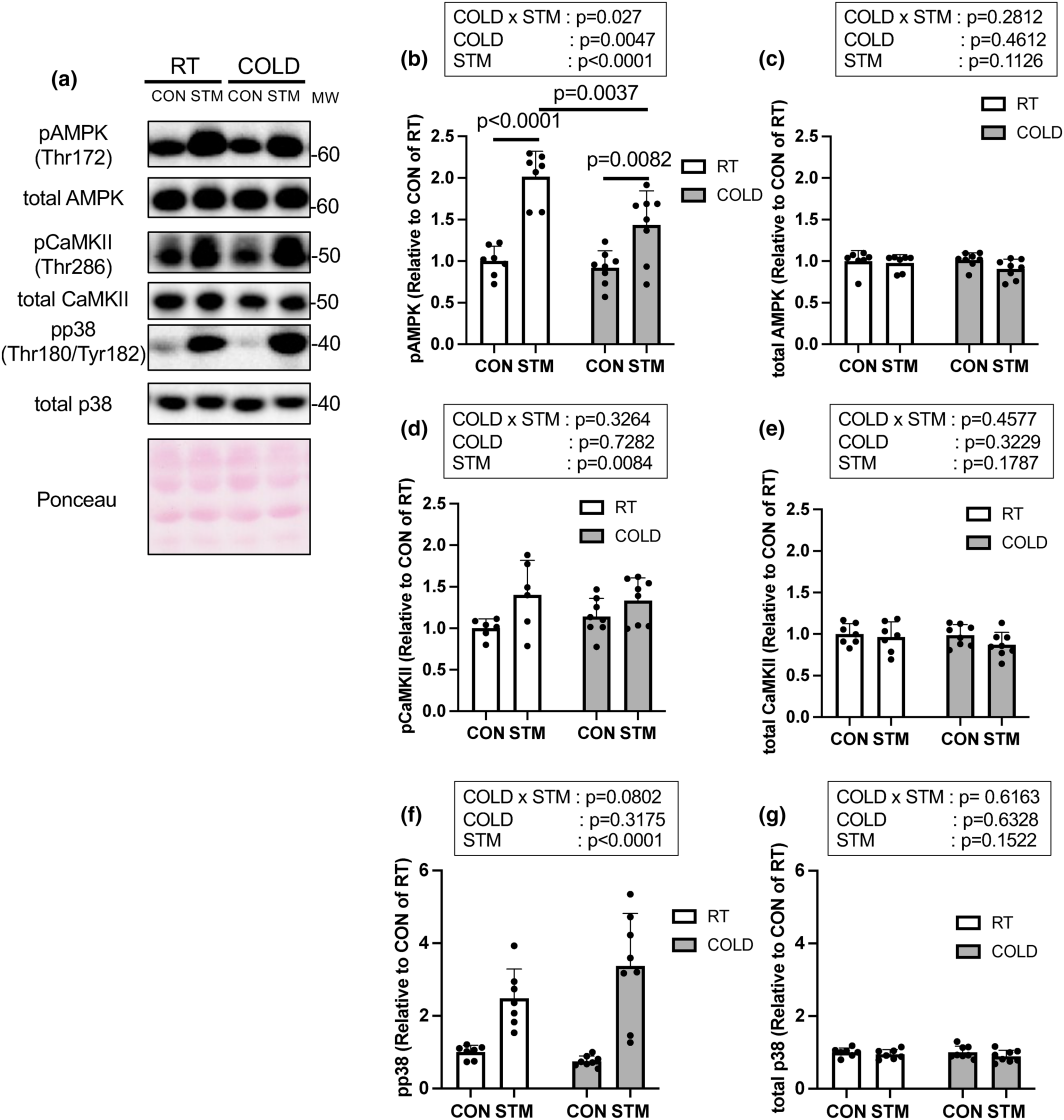
(a) Representative images of each specific protein and ponceau staining. (b) Phosphorylated and (c) total AMP‐activated kinase (AMPK), (d) Phosphorylated and (e) total calcium calmodulin‐dependent protein kinase II (CaMKII), (f) Phosphorylated and total p38 immediately after the last muscle contraction. *n* = 6–8 muscles, Data are mean ± SD.

Representative band images are shown in Figure 5a. For phosphorylated AMPK level, a significant main effect of COLD (*p* = 0.0047), STM (*p* < 0.0001), and a significant interaction of those factors (*p* < 0.027) were detected in the two‐way ANOVA. Phosphorylated AMPK level was significantly increased after the last muscle contraction in both groups (Figure 5b, RT: *p* < 0.0001, +101.6%, COLD: *p* = 0.0082, +48.7%) and the increase was significantly lower in the COLD than in the RT group (*p* = 0.0037, −28.8%). Phosphorylated CaMKII level was significantly increased after the last muscle contraction in both groups (Figure 5d, *p* = 0.0084 main effect of STM, RT: +40.2%, COLD: +17.0%). Phosphorylated p38 level was significantly increased after the last muscle contraction in both groups (pp38, Figure 5f, *p* < 0.0001 main effect of STM, RT: +148.5%, COLD: +349.6%). There was no significant main effect of COLD or interaction effect for phosphorylated CaMKII and p38. Total AMPK, CaMKII, and p38 protein levels were not significantly altered with muscle contraction or cold treatment (Figure 5c,e,g). We also analyzed phosphorylated/total protein levels of AMPK, p38, and CaMKII. The ratios were similar trend to the data without standardization by each total protein level (Figure S1).

### 3.5 *PGC‐1α* and *VEGF* mRNA expression

**FIGURE 6:**
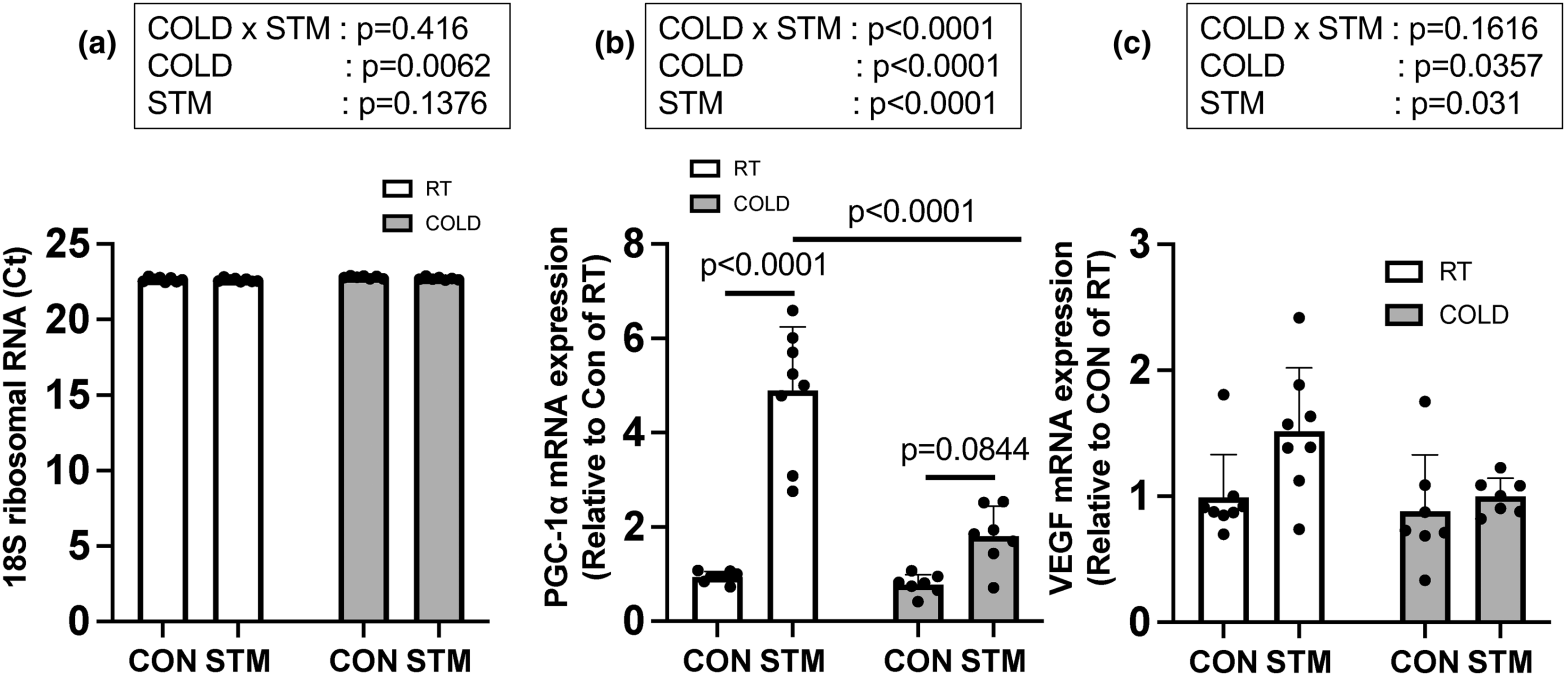
(a) 18S ribosomal RNA Ct values and, (b) *PGC*‐*1α* and (c) *VEGF* mRNA expressions 3 h after the last muscle contraction. *n* = 7 or 8 muscles, Data are mean ± SD.

Ct values of 18S ribosomal RNA that we used as an internal control were consistent among all groups (Figure 6a). Because of the high consistency of the data, there was a significant main effect of COLD but the difference is negligible (*p* = 0.0062, CON: +0.68%, STM: +0.38%). For *PGC‐1α*, a significant main effect of COLD (*p* < 0.0001), STM (*p* < 0.0001), and a significant interaction of those factors (*p* < 0.0001) were detected in the two‐way ANOVA. *PGC‐1α* mRNA expression increased after the muscle contraction only in the RT group (Figure 6b, *p* < 0.0001, +419.4%) and was significantly lower in the COLD than in the RT group after the muscle contraction (*p* < 0.0001, −63.0%). *VEGF* mRNA expression was significantly increased after the muscle contraction in both groups (Figure 6c, *p* = 0.031 main effect of STM, RT: +53.0%, COLD: +13.4%) and was significantly decreased with the cold treatment (*p* = 0.0357 main effect of COLD, RT: −11.0%, COLD: −34.0%). When *PGC‐1α* and *VEGF* mRNA expressions were calculated by another internal control, beta‐2‐microglobulin, their expressions were the same trend as the data standardized by 18S ribosomal RNA (Figure S2).

## 4 DISCUSSION

We examined the effects of local cooling on *PGC‐1α* and *VEGF* mRNA expressions and their upstream metabolic responses and signaling molecules after muscle contraction. The following are the study's main results. Muscle contraction with local cooling (1) attenuated the post‐contraction increase in *PGC‐1α* mRNA expressions; (2) attenuated muscle contraction‐induced phosphorylation of AMPK but not p38 or CaMKII; (3) reduced the integral of >50% of 100 Hz tension, which implies the volume of high‐intensity muscle contraction, and lowered the amount of muscle glycogenolysis and lactate accumulation.

Muscle contraction with local cooling, which lowered to 19.42 ± 0.44°C of muscle temperature than contraction with 30.57 ± 0.58°C at the end of the experiment, attenuated the increase in *PGC‐1α* mRNA expression compared to normothermic conditions. Cooling significantly decreased *VEGF* mRNA expressions in control and stimulated legs but did not affect the contraction‐induced increase of *VEGF*. These findings are in line with a previous study that depicted that 29°C of muscle temperature and 20°C of skin temperature during exercise inhibited the increase in *PGC‐1α*, but not *VEGF*, mRNA expression after cycling for 1 h in humans (Meister et al., 2021). However, they did not measure the activity of the upstream signaling molecules, and the mechanism was unidentified. Three main signaling molecules are involved with *PGC‐1α* mRNA expressions namely AMPK, CaMKII, and p38 (Perry & Hawley, 2018; Zhang et al., 2014). In the current study, low muscle temperature attenuated the phosphorylation of AMPK, but not CaMKII or p38, after contraction. Indeed, *PGC‐1α* mRNA expression in the activated muscle during contraction is, at least in part, an AMPK‐related mechanism (Terada et al., 2002). AMPK‐induced phosphorylation of PGC‐1α is necessary for mitochondrial biogenesis after AMPK pharmacological activation (Jäger et al., 2007). These results suggest that the attenuation of *PGC‐1α* mRNA expressions would be linked to a decrease in AMPK phosphorylation. However, muscle contraction‐induced increases in p38 and CaMKII phosphorylations were not altered significantly with cooling in this study. Although the magnitude of increased *PGC‐1α* mRNA level was attenuated, *PGC‐1α* mRNA expression tended to be increased after the muscle contraction in the COLD group (*p* = 0.08). Since AMPK is not only a factor that increases *PGC‐1α*, p38 and CaMKII phosphorylations may be associated with the increase in *PGC‐1α* mRNA expression in the COLD condition after muscle contraction.

What is attributable to the decrease in AMPK phosphorylation after muscle contraction in this study? We consider that a decrease in the volume of high‐intensity muscle contraction (the integral of >50% of 100 Hz tension) would be ascribed to the decrease in AMPK phosphorylation. AMPK is activated by changes in the intramuscular [ADP][AMP]:[ATP] ratio and lower‐intensity exercise leads to lower intracellular free ADP and AMP concentrations (Chen et al., 2003). Indeed, AMPK phosphorylation has been demonstrated to increase in an exercise intensity‐dependent manner (Chen et al., 2003), and exercise intensity influences *PGC‐1α* mRNA expression rather than exercise duration (Egan et al., 2010). Additionally, in our study, immediately after muscle contraction, glycogen concentration was higher and lactate concentration was lower in the COLD group compared with the RT group. These results denote that low muscle temperature attenuated the amount of glycogen degradation during muscle contraction. A significant negative correlation is observed between muscle glycogen concentration following exercise and the activity of AMPK, and a significant positive correlation between the amount of muscle glycogen reduction and the activity of AMPK (Rothschild et al., 2022). Taken together, low muscle temperature decreased high‐intensity contraction volume and muscle glycogen breakdown, which may have reduced contraction‐induced phosphorylation of AMPK.

The results regarding glycogen concentration and lactate accumulation differed from those of the previous study. In a study in which rat TA was subjected to isometric contraction, no significant difference was found between decreases in glycogen concentration after the contraction at different muscle temperatures (28 and 36°C) (Blomstrand et al., 1985). Maximum tetanic tension was not affected by the difference in muscle temperature in the study. This discrepancy from our study may be due to the different muscle temperatures used in their study (28 and 36°C) and our study (19 and 30°C) because it has been reported that isometric maximum tetanic tension was 99%, 95%, and 86% relative to 35°C at 30, 25 and 20°C, respectively, of muscle temperature (Ranatunga, 1980). On the other hand, in a human study using voluntary isometric contraction, the endurance time to maintain the tension at 2/3 maximal voluntary contraction tended to be shorter at 22.5°C than at 32.6°C of muscle temperature (Edwards et al., 1972). At the time, the muscle lactate concentrations after the first contraction were 45.5 and 60.3 μmol/g dry muscle at muscle temperature 22.5 and 32.6°C, respectively. In our study, maximum tension and the integral of >50% of 100 Hz tension at 20 Hz were decreased by 9.7% and 78%, respectively, with cooling. This result suggests that low temperature (approximately 20°C) reduced high‐intensity contraction volume in isometric contractions. Therefore, the decrease in the volume of high‐intensity muscle contraction is associated with the decline in these metabolic responses such as glycogen degradation during muscle contraction with low muscle temperature.

We assume that the reduction in maximal muscle tension and the volume of high‐intensity muscle contraction with low muscle temperature causes low metabolic responses, AMPK phosphorylation, and mRNA expressions of *PGC‐1α* following muscle contraction. This low muscle force accompanied by low temperature was consistent with the previous studies using rodent muscles. A classic study by Truong et al. revealed a linear decrease in the maximal isometric tetanic tension stimulated by electrical stimulation from 40 to 20°C using the triceps muscle of rats (Truong et al., 1964). Another experiment using single fibers from the mouse flexor brevis revealed that tetanic maximum force response to electrical stimulation at 20°C decreased by 20% compared with at 32.5°C (Lännergren & Westerblad, 1987). Additionally, it is demonstrated that the extensor digitorum longus, which has a predominant fast‐twitch muscle, exhibits a greater reduction in maximum tension following Ca^2+^ activation than the soleus muscle at 22°C compared to 35°C (Stephenson & Williams, 1981). Since the TA muscles of rats used in this study have mostly Type II fibers (Kitaoka et al., 2019), the effect of low temperature on muscle tension was observed clearly in this study.

*PGC‐1α* and *VEGF* are genes involved in mitochondrial biogenesis and in angiogenesis, respectively. In previous studies, it has been noted that cooling following exercise accelerated postexercise increases in *PGC‐1α* and *VEGF* gene expressions, hence enhancing the effects of exercise on muscle oxidative metabolism (Ihsan et al., 2014; Joo et al., 2016; Slivka et al., 2013). Indeed, repeated post‐exercise cold (15°C) water immersion for 15 min enhances mitochondrial biogenesis at the transcription level in human skeletal muscles following 4 weeks of endurance training (Ihsan et al., 2015). There was only a limited effect of local muscle cooling on the transcriptional response related to mitochondrial biogenesis at rest (Robins et al., 2022; Zak et al., 2017) and local muscle cooling after exercise did not affect the expression of mitochondrial biogenesis‐related genes compared to recovery from exercise in control conditions (Shute et al., 2017). Nevertheless, the current research demonstrated that contraction with cooling muscles to approximately 20°C attenuates the post‐contraction *PGC‐1α* and *VEGF* mRNA expressions. Therefore, these findings suggest that low muscle temperature, accompanied by declined muscle performance, may reduce the muscle adaptation involved with oxidative metabolism to long‐term exercise training. To avoid this effect of cooling, it is critical to maintain muscle temperature during exercise with thermal clothing and warm‐up exercise. We further evaluate the effects of chronic muscle contraction with cooling on mitochondrial biogenesis and angiogenesis in a future study.

In this study, low temperature inhibited contractile performances namely contraction and relaxation velocities and maximum muscle tension but the mechanisms could not be clarified. The increased Ca^2+^ sensitivity from 15 to 30°C is observed in skinned single muscle fibers (Debold et al., 2006). This may be corroborated by the observation using the rat‐skinned fiber experiment that elevated temperature enhanced Troponin C affinity for Ca^2+^, thereby facilitating myosin binding to actin but the temperature ranged from 10 to 22°C (Sweitzer & Moss, 1990). The slowed relaxation rate may be related to the suppression of sarco(endo)plasmic reticulum (SR) Ca^2+^‐ATPase (SERCA) activity because the SERCA activity was decreased by low temperature (below 20°C) (Dode et al., 2001). These findings suggest that the intracellular Ca^2+^ accumulation may occur during and/or after muscle contraction in lower temperatures but it has not been examined yet. Since Ca^2+^ is an important signaling molecule that affects the transcription of various genes (Chin, 2004), it is necessary to investigate how changes in Ca^2+^ concentration after contraction with local cooling affect gene expressions in various pathways.

A limitation is that this study did not demonstrate a direct relationship between AMPK and *PGC‐1α* mRNA expression using knockout animal or siRNA experiments. Another study also found that local cooling weakened exercise‐induced *PGC‐1α* mRNA expression but there were no significant differences in power outputs (Meister et al., 2021), suggesting that AMPK might not be altered with the cooling in their study. Jørgensen et al. found that AMPK α1 and α2 isoforms are not essential for the acute exercise‐induced *PGC‐1α* mRNA expression by the study used AMPK α1 and α2 each KO mice (Jørgensen et al., 2005). Tadaishi et al. reported that treadmill exercise increased *PGC‐1α* mRNA in mouse skeletal muscle via beta2‐adrenergic receptor activation but not AMPK activation (Tadaishi et al., 2011). Although we consider that AMPK is associated with this inhibition of *PGC‐1α* mRNA response after muscle contraction, the mechanisms should be further elucidated.

## 5 CONCLUSION

We observed that muscle contraction with local cooling attenuated the post‐contraction increases in *PGC‐1α* mRNA expressions possibly via low AMPK phosphorylation. This decrease in AMPK phosphorylation was ascribed to reductions in the volume of high‐intensity muscle contraction, muscle glycogenolysis, and lactate accumulation owing to local cooling. This study suggests that low muscle temperature, which is accompanied by reduced muscle performance, may diminish the muscle adaptation involved with oxidative metabolism to long‐term exercise training.

## AUTHOR CONTRIBUTIONS

Daisuke Hoshino, Ryo Takagi, and Yutaka Kano conceptualized and designed the research; Daisuke Hoshino, Ryota Wada, Yutaro Mori, Ryo Takeda, and Yudai Nonaka performed the experiments; Daisuke Hoshino, Ryota Wada, and Yutaro Mori analyzed the data; Daisuke Hoshino, Ryota Wada, Yutaro Mori, Ryo Takeda, Yudai Nonaka, Ryotaro Kano, Ryo Takagi, and Yutaka Kano interpreted the results of experiments; Daisuke Hoshino, Ryota Wada, and Yutaro Mori prepared the figures; Daisuke Hoshino, Ryota Wada, and Yutaro Mori drafted; Daisuke Hoshino, Ryota Wada, Yutaro Mori, Reo Takeda., Yudai Nonaka, Ryotaro Kano, Ryo Takagi, and Yutaka Kano edited, and revised the manuscript and approved the final version of the manuscript.

## CONFLICT OF INTEREST STATEMENT

No conflicts of interest, financial, or otherwise are declared by the authors.

## ETHICS STATEMENT

All experiments were approved by the University of Electro‐Communications Institutional Animal Care and Use Committee (Approval No. A39).

## Supporting information


Figure S1.

Figure S2.

## Data Availability

The data that support the findings of this study are available from the corresponding author upon reasonable request from a qualified researcher.
