# Supplementary material for: Cooling of male rat skeletal muscle during endurance‐like contraction attenuates contraction‐induced PGC‐1α mRNA expression
Source: Physiol Rep. 2023 Nov 14;11(21):e15867. doi: 10.14814/phy2.15867 (PMC10644292; doi:10.14814/phy2.15867)
Supplement: Supplementary file 1 — Figure S1. [file PHY2-11-e15867-s001.docx]

Figure S1 (A) pAMPK/total AMPK, (B) pCaMKII/total CaMK and (C) pp38/total p38 protein levels after the last bout of muscle contraction. n = 7 or 8 muscles, Data are mean ± SD.
