# Supplementary material for: Cooling of male rat skeletal muscle during endurance‐like contraction attenuates contraction‐induced PGC‐1α mRNA expression
Source: Physiol Rep. 2023 Nov 14;11(21):e15867. doi: 10.14814/phy2.15867 (PMC10644292; doi:10.14814/phy2.15867)
Supplement: Supplementary file 2 — Figure S2. [file PHY2-11-e15867-s002.docx]

Figure S2 (A) beta-2-microglobulin RNA Ct values and, (B) *PGC-1α* and (C) *VEGF* mRNA expressions 3 h after the last muscle contraction. *PGC-1α* and *VEGF* were normalized by beta-2-microglobulin. n = 6-8 muscles, Data are mean ± SD.
